# NGF, Brain and Behavioral Plasticity

**DOI:** 10.1155/2012/784040

**Published:** 2012-02-16

**Authors:** Alessandra Berry, Erika Bindocci, Enrico Alleva

**Affiliations:** Section of Behavioral Neurosciences, Department of Cell Biology and Neurosciences, Istituto Superiore di Sanità, Viale Regina Elena 299, 00161 Rome, Italy

## Abstract

Nerve Growth Factor (NGF) was initially studied for its role as a key player in the regulation of peripheral innervations. However, the successive finding of its release in the bloodstream of male mice following aggressive encounters and its presence in the central nervous system led to the hypothesis that variations in brain NGF levels, caused by psychosocial stressor, and the related alterations in emotionality, could be functional to the development of proper strategies to cope with the stressor itself and thus to survive. Years later this vision is still relevant, and the body of evidence on the role of NGF has been strengthened and expanded from trophic factor playing a role in brain growth and differentiation to a much more complex messenger, involved in psychoneuroendocrine plasticity.

## 1. Introduction

At the beginning of the 1950s, the Nobel Laureate Rita Levi-Montalcini discovered and characterized the Nerve Growth Factor (NGF) as a key player in target-mediated regulation of peripheral innervations [1, 2]. The pioneer studies performed by her and Victor Hamburger first showed that the nervous system requires an adequate supply of trophic factors (neurotrophins—NTs) from the environment for survival and development. In addition, and most intriguingly, these studies first raised the concept of cell death playing a pivotal role in this specific context. In particular, target tissues might determine the size and morphology of the peripheral innervations by regulating cell death among neurons and by making the NTs available only in limited amounts, finally resulting in the selection only of those neurons which have established the quantitative and qualitative numbers of connections (with the target tissue). Subsequent studies have demonstrated that, during nervous system development, NGF is released by the target tissue, taken up in responsive neurons by receptor-mediated endocytosis and transported retrogradely to the cell body where it exerts its trophic/differentiative effects through its low (p75NTR) and high affinity receptors (TrkA) [3–7]. In particular, while the p75NTR can cause apoptosis in a variety of systems, when coexpressed with TrkA, it can modify their ligand-binding activity, dose-responsiveness, and kinase activity, leading to increased survival, neurite outgrowth, and synaptic plasticity [8–12].

Since these first investigations on target-controlled neuronal survival, NGF has been one of the most thoroughly studied NTs, regulating the survival, development, and trophism of specific neuronal populations in the peripheral and central nervous system (CNS) [13]. While in the periphery this NT was first recognized for its action on the sympathetic ganglia, in the adult brain the highest levels of NGF are found in hippocampus, cortex, and olfactory regions, which represent targets for basal forebrain cholinergic neurons [14, 15]. NGF acts as a trophic factor for these neurons since its administration in vivo increases the levels of choline acetyltransferase [16, 17] while rescuing from death basal forebrain neurons following transection of the septo-hippocampal pathways [18]. In addition to the nervous system, NTs exert their effects on various other tissue compartments [19–26]. The largest amount of NGF is produced in the salivary glands of adult male mice, which are the largest and best available source of this NT (smaller concentrations of this NT can be found in snake venom, guinea pig prostate, the seminal fluid of guinea pigs and bulls, in the human skin, and in numerous tissues and body fluids [13]). Furthermore, a number of other cells outside the nervous system, including epithelial cells, fibroblasts, lymphocytes, and activated macrophages, synthesize NGF [27–29].

Since NGF discovery, no explanation was available for almost three decades on NGF physiological role in adult animals. Most neurobiologists, for a very long time, explained its presence in adult animals because of its ancestral physiological role during ontogeny. NGF was originally described as a protein exclusively acting on sympathetic peripheral neurons and only much later it was found to be also active on central nervous system neurons and on a variety of immune cell types [30]. Overall, the presence of NGF in those limbic areas of the CNS involved in mood and cognition and in the orchestration of neuroendocrine responses and circadian activities, as well as in cells of the immune and endocrine system, indicates a much wider role for this NT than previously hypothesized and suggests that it might function as intercellular messenger or even a humoral factor to help regulate endocrine responses to stress [19, 31–35].

## 2. Stress Renewed CNS and Adrenal Plasticity

Stress may be defined as any change of the internal or external milieu perturbing the maintenance of homeostasis of an organism. In complex organisms, stress responses involve a coordinated set of intercellular signals eventually resulting in the removal of the organism from, or adaptation to, the stressful situation. Coping strategies are therefore important components of the stress response allowing an animal to “fright, fight or flight” [36]. Pivotal to these allostatic/adaptive responses is the neuroendocrine system that employs neuropeptides and hormones as mediators. The hypothalamic-pituitary-adrenal (HPA) axis (which releases the glucocorticoids—GCs—hormones) and sympathetic adrenomedullary systems (which releases catecholamines) coordinate the stress response. Although the cascade of responses to acute stressful events originates at the CNS level, peripheral structures, such as the adrenal glands, act in interplay and feedback on the brain to maintain body homeostasis [37, 38]. By contrast, chronic stress can promote and exacerbate pathophysiological conditions through these same systems, leading to allostatic overload [39]. The adaptive plasticity of chronic stress involves many mediators, including GCs, excitatory amino acids, and NTs, such as NGF and also Brain-Derived Neurotrophic Factor (BDNF). These latter can participate in the HPA axis response to stressful stimuli at different anatomical levels [38, 40].

In the wild, adult male mice exhibit high levels of intraspecific aggressive behavior towards conspecifics mainly related to territorial defense. In the laboratory setting, the agonistic behavior of male mice can be elicited after 4–6 weeks of social isolation and represents a psychosocial stressful condition that has been shown to markedly alter NGF and BDNF levels both in plasma as well as in selected brain areas, including the hypothalamus and hippocampus [41, 42]. In the mouse, NGF is mainly synthesized and released from the submaxillary salivary glands [5, 43]. Neurobehavioral studies demonstrated that both male intraspecific fighting and lactating females nest-defense, raised against a potentially infanticidal conspecific male, induce a massive release of NGF from salivary glands into the bloodstream of adults. In particular, circulating levels of NGF are highly correlated with the number of fighting episodes and most intriguingly to the social status achieved in fighting mice. Thus, subjects experiencing repeated defeat and submissions (i.e., a subordinate status) were characterized by a two-time increase in the amount of circulating NGF compared to attacking mice that achieved a dominant status [44, 45]. In this specific context it is worth to note that physical stressors, such as cold water, foot shocks, or restraint stress exposure, exert a minor effect on NGF release [43, 46].

**Figure 1:**
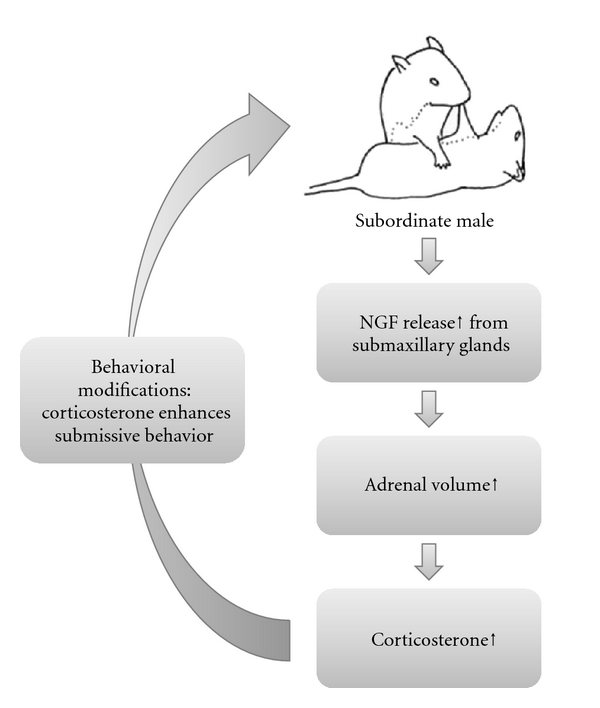
NGF secretion may exert an inhibitory feedback effect on the aggressive behavior of male mice. The higher NGF release and the hypertrophy of the adrenals, occurring in subordinates male mice, suggest a “regulative loop” involving NGF-mediated increase of glucocorticoids secretion (upon adrenals) acting to enhance a submissive profile.

Following fighting behavior, adrenal weight increases quite markedly and quickly in male mice suggesting that social/aggressive behavior might control circulating NGF release from salivary glands and that these might in turn control adrenal morphology as well as adrenal functional status [47, 48]. This hypothesis has been supported by data showing that exogenous NGF administration (i.p. delivery, for 10 consecutive days in order to mimic 10 subsequent daily fighting sessions) results in a marked adrenal gland hypertrophy [43, 47, 48]. Thus, NGF release may exert an inhibitory feedback effect on aggressive behavior. The higher NGF release and the hypertrophy of the adrenals, occurring in subordinates male mice, suggest a “regulative loop” involving NGF-mediated increase of glucocorticoids secretion (upon adrenals) acting to enhance a submissive profile (Figure 1) [46, 47, 49].

Further experimental evidence supports the idea that NGF expression may be regulated by behavioral activation. In particular, intermale aggressive behavior induces a large increase in NGF mRNA and protein in hypothalamus, especially in the paraventricular nucleus, with no measurable changes in cerebral cortex, hippocampus, and cerebellum [50]. The increase in hypothalamic NGF following intermale aggressive behavior is not abolished by sialoadenectomy, suggesting that hypothalamic NGF is not of salivary origin [43]. Alleva and Aloe hypothesized that the rather rapid increase in levels of brain NGF, which follows a psychosocial stressful event, could allow some renewal of brain plasticity at adulthood [46].

Indeed, NGF could contribute to structural changes, such as the formation of dendritic spines or collateral sprouting, ultimately altering the structure of neural connections in the mature brain [46, 47]. Another possibility is that hypothalamic NGF might affect levels of other peptides or hormones present in the hypothalamus [51–55]. Taken together, these studies suggest that hypothalamic NGF levels are responsive to (and modified by) stimuli of a psychological nature, most likely associated with anxiety and fear. As hypothalamus is involved in the maintenance of physiological homeostasis, it is likely that hypothalamic NGF affects, or cooperates with, hormones and/or neurotransmitters present in this brain area to ultimately integrate behavioral and neuroendocrine responses [31, 46].

In this complex scenario, peripheral organs, such as adrenal glands, might be seen as remote CNS stations able to regulate behavioral reactivity. Such an “extremely peripheral” location provides most of the body with a remote station, in strict hormonal connection with some functionally integrated brain zones, to easily and efficaciously control the entire organisms. In addition and more importantly, these glands are morphologically (and functionally) endowed with a very high degree of plasticity leading to profound endocrine and behavioral modifications, and both short-term changes in behavioral reactivity at the individual level and changes in mouse population structure (e.g., major behavioral changes in reactivity to social stimulation) could be expected [43, 56–59]. Thus, NGF appears to play a role in stress-mediated changes in behavioral responses, leading to imprinting-like phenomena at adulthood, in which such social stimuli suddenly become relevant and produced long-lasting behavioral alterations. In particular, it could be hypothesized that variations in the hypothalamic NGF, caused by psychosocial stressor, and the related alterations in emotionality, could be functional to the development of proper strategies to cope with the stressor itself and thus to survive. NGF could shift some still unknown brain zones backwards to an “immature-like” stage [46, 49].

## 3. The Role of NGF at the Peripheral Level

Belonging to the neuroendocrine system, the anterior pituitary gland represents an important relay station between the periphery and the CNS under the control of hypothalamic hormones and peripheral signals. The integration operated by this gland determines the extent of hormonal secretion in different physiological situation and NGF and BDNF appear to play a neurotrophic role on the pituitary and stimulate synthesis of neuropeptides. The first evidence of such a role for NTs came from studies suggesting that, following intravenous injection, NGF increased the secretion of adrenocorticotropic hormone (ACTH) as well as the concentration of GCs, and that following a psychosocial stress, a massive release of NGF occurred in the peripheral circulation, associated with increased hypothalamic NGF mRNA and protein levels [43, 60, 61].

NGF is synthesized in the granular convoluted tubules within the submandibular glands soon after puberty (much more in male than in female subjects) [5, 13]. The functional significance of the large amounts of NGF observed in the mouse submandibular glands is not fully elucidated; however it has been shown to be released into the bloodstream as a result of intraspecific aggressive behavior acting on nerve, chromaffin, mast cells, and lymphocytes [43, 61, 62]. The adrenal glands are intrinsically characterized by a high degree of plasticity from a morphological (and functional) point of view [43]. Their size varies according to short- and medium-term life stressful events, and such a morpho-functional change is mirrored by subtle and rapid central modification in the expression of the hippocampal glucocorticoids receptor (GR) system, in the excitability of hippocampal neurons, neurogenesis of the dentate gyrus, synaptogenesis in the CA1 region, and dendritic remodeling in the CA3 region, just to mention few [63]. NGF appears as key player in the ontogeny of adrenal glands as its administration induces the transformation of chromaffin cells in sympathetic nerve cells in the adrenal medulla. By contrast, injections of NGF antiserum (from prenatal day 17 to postnatal day 10 in rats) inhibit adrenal development and differentiation through a massive destruction of chromaffin cell precursors [64] overall showing the main role played by NGF in critical developmental stages [37, 38].

Thus adrenal glands can be viewed as representing the last pair of vertebral ganglia. During early (in altricial rodents late-gestational) developmental stages, thanks to a complex interaction of neurohormonal factors (including GCs and NGF, as reported just below), they do not fully differentiate as adult-like ganglia but maintain several fetal-type characteristics. Therefore, their enhanced morphofunctional plasticity is maintained through adulthood, rendering them a remote (control) neurohormonal station involved in a continuous dialog with the brain for the maintenance of body homeostasis [37, 38].

A large body of evidence suggests a link between adrenal size modification and coping strategies to face stressful events which might underlie the association often found for a pathologic increase in corticosteroid hormones and vulnerability to psychiatric disorders [46]. Since the adrenal gland appears to be one of the biological targets of NGF, one way this growth factor could exert a physiologic role is through its action on this structure. NGF administered for 6–10 consecutive days results in a dose-dependent increase in adrenal weight and volume by enlarging both the adrenal medulla and the cortex [43, 49]. At present, it is not clear whether adrenal cortex hypertrophy following NGF administration in adult mice is the result of a direct action of this NT on adrenal cortex cells or whether it is mediated through its effects on ACTH release. Interestingly, it has been shown that conditions exist in which adrenal activity is uncoupled from ACTH, such as during starvation [65], and this suggests a direct effect of NGF on the adrenals under specific conditions. Furthermore, in animal models characterized by metabolic disorders involving dysregulation of the HPA axis, such as type I diabetes, corticosterone hypersecretion appears to occur independently from a surge in ACTH levels [38, 66]. NGF might also contribute as an alternative mechanism in inducing such rapid changes in adrenal sensitivity. Changes in adrenal hormone secretion (e.g., increased corticosterone levels) resulting from an increase in NGF circulating levels could exert important effects on aggressive behavior: GCs enhance aggressive behavior in a context-dependent fashion, promoting social challenge-induced aggression without increasing aggressiveness under routine conditions in rats [67, 68]. Indeed, at present, there is no evidence of direct NGF effects on neural substrates of aggressive behavior, and the high molecular weight of the NGF protein seems to exclude the possibility that it could cross the blood-brain barrier, although a possible breakage of the NGF protein into fragments acting on the CNS can be postulated [69]. From a clinical application point of view, it appears interesting to point out that successful transport of biologically active NGF across the blood-brain barrier has been achieved by covalently linking it to an antitransferrin receptor antibody or by ocular delivery [38, 70, 71]. Likewise, Aloe and Alleva, working in the team of Levi-Montalcini, analyzed all the evidences collected up to that point on the physiological activities exerted by NGF on CNS and non-CNS neurons [31]. In those years, starting with mast cells [72, 73], and much later memory B lymphocytes [74], evidences were provided for a role of NGF on psychoneuroimmune regulation [31, 46].

## 4. The Role of NGF at the CNS

Most of the studies on NGF were originally performed by Rita-Levi Montalcini on the sympathetic ganglia explanted from chick embryos [5, 75], leading to an initial fascinating but incomplete vision of NGF as a protein molecule exerting specific—although limited—effects on peripheral sympathetic neurons. Further studies, carried out, rather independently by Barde and Thoenen, and Mobley, characterized NGF effects on cholinergic neurons at the CNS level, opening the way to several lines of research aimed at understanding NGF physiological roles in the brain [7, 12, 76].

NGF is essential for the development and maintenance of sensory neurons, and for the formation of central pain circuitry, exogenous administration of this neurotrophin to rodents resulting in the rapid onset of hyperalgesia [69]. Together with its receptors (p75 and TrkA), it plays a critical trophic role on forebrain cholinergic neurons (FCNs) that degenerate during brain aging and neurodegenerative disorders [77, 78]. During development, both NGF and BDNF regulate naturally occurring cell death, synaptic connectivity, fiber guidance, and dendritic morphology [79]. Furthermore, they participate to brain plasticity, being involved in activity-dependent neuronal functions [34, 79–81]. During early postnatal development, especially during critical periods, large changes in the connectivity and organization of neural networks take place [82, 83]. In this period, the developing CNS is particularly sensitive to external stimuli, and NGF and BDNF play a key role in modulating brain plasticity to better cope with environmental stimuli. As an example, mice reared in a communal nest (CN, which consists in a single nest where three mothers keep their pups together and share care-giving behavior from birth to weaning) are characterized by increased NGF and BDNF levels in selected brain areas, including hippocampus and hypothalamus, and prolonged survival of newly generated cells in the hippocampus. These features possibly underly the higher propensity of CN mice to interact socially and their better social skills when compared to subjects reared in standard laboratory conditions [84, 85]. The adult brain still shows significant plasticity: for example, repeated agonistic intermale fighting in aged mouse selectively affects neurotrophin production. In particular, the social stress of being subordinate is able to increase NGF levels in the Subventricular Zone (SVZ) and hippocampus (a brain area involved in learning and memory processes) leading to the hypothesis of a regulatory role of NGF in the “emotional” status caused by psychosocial stressors and the physiological needs of the organism to “remember” the events leading to an appropriate coping with the stressor itself [41, 47]. Moreover, aggressive behavior can enhance the number of Ki67-positive cells in the SVZ of aged animals. This suggests that, in the aged mouse, fighting behavior may increase neurogenesis, most probably throughout proliferation and/or differentiation of brain stem cells [86], possibly contributing to reduce the neuronal damage and loss caused by prolonged GC exposure as a consequence of the social stress related to fighting [87, 88]. In addition, within the brain, the major increase in NGF following fighting has been observed in the hypothalamus a brain area involved in the activation of certain behaviors and in physiological modifications inducing changes in bodily homeostasis, suggesting a role for NGF in coping and neuroendocrine mechanisms [31].

A correlation between changes in NGF levels and anxiety-like behaviors has been observed in humans. In particular, NGF levels were found to be increased in the blood of young soldiers experiencing their first parachute jump, implying that the release of NGF in the bloodstream is triggered by the stress of the novel and highly arousing experience [62]. The key role of NGF in anxiety conditions is also suggested by findings demonstrating that alcohol or heroin withdrawal in human is also associated to changes in blood NGF levels [89].

Chronicity of stress system activation leads to the syndromal state that in 1936 Selye described as the “general adaptation syndrome” [90]. When facing a stressful situation, all physiological changes, related to coping strategies, lead both to protective and damaging effects on the body. In particular, on a short run they are essential for adaptation, maintenance of homeostasis, and survival (allostasis is maintaining stability through changes). Yet, over longer time intervals, they impose a cost (allostatic load) that can accelerate disease processes or participate to pathological changes possibly contributing to the development of neurodegenerative disorders and/or to precipitate psychopathological conditions [91].

NGF, both in the bloodstream and in certain brain areas, is differentially expressed following environmental stressors. A large number of evidence show that chronic stress can lead to anxiety- and depression-like behaviors and may influence the distribution of NGF, both in animal models and in humans [46, 92]. Reduced NGF and BDNF signaling in the adult brain may be involved in the pathophysiology of psychiatric disorders, such as depression [38, 81, 93–96], and a role for NGF has been proposed in the etiopathogenesis of schizophrenia [97]. 

NTs themselves do not directly affect mood but rather play an important functional role in the modulation of networks determining how plastic changes influences mood [94]. In fact, it has been hypothesized that, by activating NTs systems, successful antidepressant treatments and cotreatments promote activity-dependent neuronal plasticity, possibly inducing proliferative or survival effects on neural stem cells [81]. Because of the fundamental role played by these neurotrophic factors in shaping brain function, pathological alterations in their concentration or action early during development could exert long-term effects on synaptic plasticity, impairing the ability of the organism to cope with novel/stressful situations, leading to psychopathology [98]. During development, the expression of NGF and BDNF has been localized to the hippocampus and prefrontal cortex, two regions which are well-studied sites of both developmental and adult synaptic plasticity and playing a key role in psychiatric disorders [15, 99]. 

Stress during prenatal and early postnatal life may result in altered brain development and lead to a persistent sensitization of limbic circuits to even mild stress at adulthood, forming the basis for a greater susceptibility for mood and anxiety disorders (Levine, 1967 no.49; Maccari, 2003 no.117). Numerous studies performed in rodents have indicated that NTs are sensitive to the stress of maternal separation and to changes in the rearing conditions and that environmental stimulation can have both short- and long-term effects on NTs levels [84, 100–107]. It is possible to hypothesize that while milder manipulations could promote neural plasticity, chronic stressful conditions could sensitize limbic structures to stress, decreasing brain plasticity and leading to higher susceptibility to psychopathology [102, 108]. As an example, in rodents, separating mother and infant for brief periods results in increased NGF expression in the hippocampus, cerebral cortex, and hypothalamus in a time-dependent manner [101, 104] while longer periods of maternal separation (24 h) also result in increased rate of cell death in the neocortex, white matter, and granule cells of the dentate gyrus in 12-day-old rats [109]. Following chronic communal nesting increased BDNF levels in association with reduced neurogenesis and increased depression-like behavior have been also found in CD-1 mice [84].

Epidemiological studies indicate that anxiety and mood disorders are characterized by sex differences in prevalence, presentation or therapeutic outcomes. A recent study suggests that epigenetic changes, presumably in the male germ cells, might alter the exposure to the hormones that mediate brain masculinization, providing insight into the heritability and pathophysiology of sex-biased neurodevelopmental disorders [110]. In addition, a very interesting work from Cirulli and collaborators provides evidence for a specific role of NTs in such a gender-specific vulnerability. In particular, BDNF and NGF were identified as neuroendocrine markers underlying differential responses to maternal deprivation in males and females rhesus macaques. The selective changes in BDNF levels in females suggest a possible mechanism for the greater vulnerability to mood disorders of this gender as reported in humans [103].

The importance of early life experiences in the etiology of psychiatric disorders has been now recognized; however it has been often underemphasized. Early life stressful events, such as childhood trauma and neglect, are associated with depression and anxiety disorders and sustained changes in the HPA axis [111–113]. These associations strengthen the hypothesis that environmental factors during development could lead to long-term enduring changes in the set-point of the neuroendocrine system physiology and emotional behavior.

Changes in the levels of NGF in the CNS appear also to play an important role in the context of neurodegenerative disorders such as Alzheimer disease (AD). The disruption of NGF gene in transgenic mice leads to a lethal phenotype; thus only the study of phenotypic knockout of NGF at adulthood is possible [114]. Transgenic mice expressing a neutralizing anti-NGF recombinant antibody are characterized by an age-dependent neurodegenerative pathology including amyloid plaques, insoluble and hyperphosphorylated tau, and neurofibrillary tangles in cortical and hippocampal neurons. Furthermore, they show an extensive neuronal loss throughout the cortex, cholinergic deficit in the basal forebrain in addition to behavioral deficits, overall being strikingly reminiscent of human AD [115]. NGF precursor (pro-NGF) has been shown to be highly expressed in the brains of AD patients and to be neurotoxic when bound in a heterotrimer with the p75 and the receptor sortilin. Interestingly, sortilin levels are increased in aged central and peripheral neurons, possibly rendering neurons more vulnerable to the age-related increases in pro-NGF [116]. More recently, Capsoni and coworkers postulated that neurodegeneration occurring in a transgenic mouse model of AD is provoked by an imbalance of proNGF/NGF and, consequently, of TrkA/p75 signaling such that the inactivation of TrkA determines a strong cholinergic deficit and AD-like neurodegeneration [117]. Thus, overall, the NGF/TrkA signaling pathway has emerged as a promising therapeutic target for the AD pathology [118].

In addition to a possible therapeutic role in the context of neurodegenerative disorders, Chiaretti and coworkers investigated the role of NGF in regard to severe traumatic brain injury (TBI) in pediatric patients [119]. TBI is the most common cause of death and acquired disability among children and young adults in developed countries and its clinical outcome depends both or the primary cerebral lesions and or the extent of secondary brain damage which involves neuroinflammatory mechanisms. It has been suggested that interleukin-6 might participate to the mechanisms of recovery from TBI through the modulation of NGF biosynthesis, elevated levels of this neurotrophin representing a reliable marker of good prognosis, at least in children [119]. Moreover, and more recently, the same authors proposed that NGF might represent an effective and safe adjunct therapy in patients (children and adults) with severe hypoxicischemic injury suggesting a neuroprotective mechanism exerted by NGF on the residual viable neurological pathways of these patients [120].
